# Lopinavir/Ritonavir versus Lamivudine peri-exposure prophylaxis to prevent HIV-1 transmission by breastfeeding: the PROMISE-PEP trial Protocol ANRS 12174

**DOI:** 10.1186/1471-2334-12-246

**Published:** 2012-10-06

**Authors:** Nicolas Nagot, Chipepo Kankasa, Nicolas Meda, Justus Hofmeyr, Cheryl Nikodem, James K Tumwine, Charles Karamagi, Halvor Sommerfelt, Dorine Neveu, Thorkild Tylleskär, Philippe Van de Perre

**Affiliations:** 1 INSERM U 1058 and CHU, Montpellier, France; 2 Universite Montpellier 1, Montpellier, France; 3 Département de Bactériologie-Virologie et Département d’Information Médicale, CHU Montpellier, Montpellier, France; 4 Department of Paediatrics and Child Health, University of Zambia, School of Medicine, Lusaka, Zambia; 5 Centre of International Research for Health, Faculty of Health Sciences, University of Ouagadougou and Centre MURAZ, Bobo-Dioulasso, Burkina Faso; 6 University of Western Cape, Cape Town, South Africa; 7 Department of Paediatrics and Child Health, College of Health Sciences, School of Medicine Makerere University, Kampala, Uganda; 8 Centre for International Health, University of Bergen, Bergen, Norway; 9 Division of Infectious Disease Control, Norwegian Institute of Public Health, Oslo, Norway

## Abstract

**Background:**

Postnatal transmission of HIV-1 through breast milk remains an unsolved challenge in many resource-poor settings where replacement feeding is not a safe alternative. WHO now recommends breastfeeding of infants born to HIV-infected mothers until 12 months of age, with either maternal highly active antiretroviral therapy (HAART) or peri-exposure prophylaxis (PEP) in infants using nevirapine. As PEP, lamivudine showed a similar efficacy and safety as nevirapine, but with an expected lower rate of resistant HIV strains emerging in infants who fail PEP, and lower restrictions for future HIV treatment. Lopinavir/ritonavir (LPV/r) is an attractive PEP candidate with presumably higher efficacy against HIV than nevirapine or lamivudine, and a higher genetic barrier to resistance selection. It showed an acceptable safety profile for the treatment of very young HIV-infected infants. The ANRS 12174 study aims to compare the risk of HIV-1 transmission during and safety of prolonged infant PEP with LPV/r (40/10 mg twice daily if 2-4 kg and 80/20 mg twice daily if >4 kg) versus Lamivudine (7,5 mg twice daily if 2-4 kg, 25 mg twice daily if 4-8 kg and 50 mg twice daily if >8 kg) from day 7 until one week after cessation of BF (maximum 50 weeks of prophylaxis) to prevent postnatal HIV-1 acquisition between 7 days and 50 weeks of age.

**Methods:**

The ANRS 12174 study is a multinational, randomised controlled clinical trial conducted on 1,500 mother-infant pairs in Burkina Faso, South Africa, Uganda and Zambia. We will recommend exclusive breastfeeding (EBF) until 26th week of life and cessation of breastfeeding at a maximum of 49 weeks in both trial arms.

HIV-uninfected infants at day 7 (± 2 days) born to HIV-1 infected mothers not eligible for HAART who choose to breastfeed their infants.

The primary endpoint is the acquisition of HIV-1 (as assessed by HIV-1 DNA PCR) between day 7 and 50 weeks of age. Secondary endpoints are safety (including resistance, adverse events and growth) until 50 weeks and HIV-1-free survival until 50 weeks.

**Discussion:**

This study will provide a new evidence-based intervention to support HIV-1-infected women not eligible for HAART to safely breastfeed their babies.

**Trial registration number ( http://www.clinicaltrials.gov):**

NCT00640263

## Background

Out of the 420,000 annual new paediatric infections of HIV-1, more than 200,000 are a result of breast milk transmission, almost exclusively in developing countries. By pooling data from five observational studies, the estimated additional risk of transmission attributable to breastfeeding is 14% (95% CI: 7-22%) [1]. In recent years, observational studies including prospective cohorts have shown that exclusive breastfeeding (EBF) is associated with a reduced risk of HIV-1 transmission as compared to mixed feeding [2-5].

Postnatal transmission of HIV-1 through breast milk remains an unsolved challenge in many resource-poor settings. In Sub-Saharan Africa, especially in the rural areas, replacement feeding has proven a problematic alternative to breastfeeding because of social, cultural, economic and hygienic constraints [6]. Moreover, exclusively or predominantly breastfed infants are likely to have a substantially lower risk of succumbing to common childhood infections such as diarrhoea and pneumonia [7,8] that also inflict a substantial nutritional insult. Therefore, strategies that both prevent MTCT and allow for optimal breastfeeding are urgently needed. One option is to reduce the infectivity of the mothers during the breastfeeding period. The recently completed “Kesho Bora” randomised controlled trial showed that HIV transmission risk using maternal HAART from 28–36 weeks pregnancy until 6 months after birth was 4.9% (95%CI: 3.1-7.6) vs. 8.4% (95%CI:6.0-11.6) using a short course ART (standard PMTCT) [9]. Another study in Botswana reported even lower transmission risks at 6 months using two different HAART regimens (2% and 1%) [10].

Another option is to give infants a prophylaxis during the breastfeeding period (peri-exposure prophylaxis-PEP). The advantages of PEP compared with maternal HAART are that: ART drug prophylaxis in an uninfected child carries no risk of selection of resistant viral strains; it may be more acceptable and applicable; it carries a lower cost and it spares mothers from using HAART at a stage of disease when its benefits have not been demonstrated, thereby avoiding HAART side effects and selection of resistant viral strains.

Several studies concurred to prove the concept that PEP can prevent postnatal transmission, using either nevirapine (NVP) or lamivudine (3TC) and various durations of PEP (Table 1). The risks of postnatal transmission of HIV at 6 months with PEP ranges from 1.2% using 3TC (95%CI: 0–2.4, among infants not infected with HIV at 6 weeks) to 1.8% using NVP (among infants not infected with HIV at one week). This strategy proved very efficacious during the period of prophylaxis, but this effect faded off after drug withdrawal as the HIV exposure continued [11,12]. All the studies used PEP for a maximum of 6 months while many HIV-infected women continued to breastfeed until 12 months.

**Table 1:** Number of events and sample size according to potential efficacy rates in both arms

		**HIV transmission in 3TC arm**		
		*0.03*	*0.04*	*0.05*
HIV transmission in LPV/r arm	0.01	*1428 subjects*	*682 subjects*	*430 subjects*
		*27 events*	*16 events*	*12 events*
	0.015	*3190 subjects*	*1223 subjects*	*704 subjects*
		*67 events*	*32 events*	*22 events*
	0.02		*2204 subjects*	*1118 subjects*
			*63 events*	*37 events*
	0.025			*1800 subjects*
				*64 events*

Tolerance of extended NVP was acceptable although 15% of babies experienced NVP-related side effects, while no drug-related severe adverse events were reported with 3TC [13]. Major concerns arose from a sub-study of SWEN in India which reported that 92% of babies infected despite NVP prophylaxis harboured viruses resistant to NVP, thus jeopardizing the use of any drug of this therapeutic class (NNRTI) for future treatment of these infants, which restricts considerably the number of ART drugs available in low-income countries. These data were expected as NVP has a low genetic barrier and is therefore very prone to select resistant strains.

Based on the results of the above studies, WHO recommends to use either maternal HAART or NVP during breastfeeding to prevent postnatal HIV transmission [14]. In addition, breastfeeding is recommended until 12 months to reduce the increased morbidity and mortality related to early weaning. However, the PEP strategy has never been evaluated for the whole duration of breastfeeding.

The choice of the ideal drug for PEP should result from a balance between efficacy, safety and resistance induced among infants who will fail. In this respect, we believe that 3TC is a better drug than NVP for PEP as it has a better safety profile (including clinical consequence of resistance in infected babies) and a presumably similar efficacy [15,16].

Very recent findings suggest that Lopinavir/Ritonavir (LPV/r), with its paediatric formulation, may be a very good candidate for HIV-1 PEP. LPV/r is one of the most potent antiretroviral drugs, with a much higher anti-HIV-1 activity than 3TC or NVP [17]. In addition, its high genetic barrier makes the development of LPV resistant strains very unlikely. Despite these very promising features for HIV prophylaxis, LPV/r was not initially considered for PEP because no safety data were reported in young infants. However, recent data suggest a good safety profile. In the CHER trial, 252 HIV-1 infected infants aged 6 to 12 weeks (median 7.2 weeks) received a combination therapy including LPV/r (300/75 mg per m^2^ bid until 6 months of age, then 230/57 mg per m^2^ bid), Zidovudine and Lamivudine [18]. During the 40-week follow-up, neutropaenia occurred in 10 children, anemia in three, and elevated aminotransferase levels in two. Four children switched from Zidovudine to Stavudine because of neutropaenia. LPV/r was not discontinued in any child and no severe adverse event was related to this drug.

A smaller cohort of 21 infants aged 7 to 26 weeks treated with LPV/r (300/75 mg/m^2^ twice daily) plus 2 NRTI confirmed these encouraging findings [19]. Tolerance was good with six grade 3 adverse events occurring in three children possibly related to HAART: asymptomatic serum/sodium disturbances in two infants that resolved with disruption of therapy for 0–2 weeks and did not recur thereafter, and alanine aminotransferase elevation that did not recur after treatment suspension for 3 days. Some minor cholesterol disturbances were recorded in eight infants. Finally, a pharmacokinetic and safety study among 10 perinatally infected infants aged 3 to 6 weeks was recently reported. Adverse events were limited to transient grade 3 neutropaenia in three subjects (2 likely related to NRTI drugs and 1 temporally related to high plasma levels of LPV/r and stabilised after restarting LPV/r at reduced dose (the dose we will use for our trial). However, one fatal case of overdosage was recorded in France (Lyon) where a young infant died after absorption of a 10-fold higher dose than recommended at his age (S. Blanche, personal communication). Therefore, cautions should be taken to educate mothers and avoid massive overdosage.

The same studies by Chadwick and coll. also provided pharmacological data on LPV/r in infants [20]. Briefly, the bioavailability of LPV/r is reduced in young infants and therefore there is a need to adapt dosing per kg throughout the first year of life. These data were confirmed by a French pharmacokinetic study among 66 neonates who received LPV/r to prevent late perinatal HIV transmission. A dosage regimen of LPV 15 mg/kg bid was recommended for this age-group to achieve therapeutic levels [21].

The primary objective of the study is to compare the HIV-1 transmission risk between 7 days and 50 weeks of age when infants are given LPV/r (40/10 mg twice daily if 2-4 kg and 80/20 mg twice daily if >4 kg) or lamivudine (12 mg twice daily if <6 kg, 24 mg per day if 6.0 to 9.0 kg, and 36 mg per day if ≥ 9.0 kg) from day 7 until one week after cessation of BF (maximum duration of prophylaxis: 50 weeks).

Secondary objectives are i) to assess the safety of long-term infant prophylaxis with LPV/r versus lamivudine (including resistance, adverse events and infant growth) until 50 weeks, ii) HIV-1-free survival until 50 weeks and iii) to build clinical trials capacity at the four study sites.

## Methods

### Study design

We designed a multicentre randomised controlled pragmatic trial. Although double blinding is not implemented for this study, we will mask drug bottles with study labels and keep study physicians in charge of study visits and adverse events collection, and staff in charge of anthropometric measurements unaware of treatment allocation. In addition, biologists in charge of infant HIV diagnosis (primary outcome) will be blinded.

We here describe the version 3.0 of the study protocol, registered at http://www.clinicaltrials.gov (NCT00640263).

**Figure 1:**
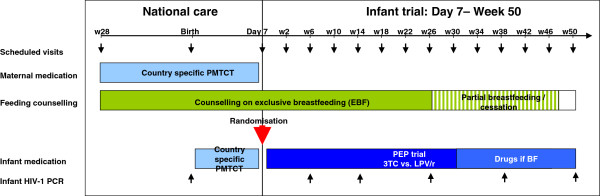
ANRS 12174 PROMISE-PEP trial design including scheduled follow-up visits.

All women who agree to participate in the study will be counselled to exclusively breastfeed their babies up to 6 months of age, and to gradually introduce complementary food thereafter and to stop breastfeeding at 49 weeks. This will give women sufficient time to prepare for cessation of breastfeeding. Continuous counselling on a nutritionally balanced diet will be made available up to 50 weeks of age. In order to cover the whole breastfeeding period, the maximum duration of prophylaxis is 50 weeks. The infants will be tested for HIV-1 at Birth, Day 7, Week 6, 14, 26, 38 and 50 (Figure 1).

### Participants

Infants will be recruited and followed in Ouagadougou (Burkina Faso), East London (South Africa), Mbale (Uganda) and Lusaka (Zambia). HIV-infected pregnant women will be identified at antenatal clinics. Breastfed infants of HAART-treated mothers ingest sufficient amounts of ART in milk to reach plasma therapeutic levels (at least for NVP, LPV/r, AZT and 3TC) [22]. Therefore, in order to avoid overdosage of LPV/r and Lamivudine in babies, our trial will only recruit babies born to mothers that are not eligible for HAART according to local recommendations, i.e. with >350 CD4 cells/μL blood.

A baby will be included if she/he:

• is a singleton

• is breastfed at day 7 by her/his mother and her/his mother intends to continue breastfeeding for at least 6 months

• has a post-partum blood sample with a negative HIV-1 DNA PCR test result at day 7 (+/− 2 days)

• has received some antiretroviral prophylaxis at birth or during the first week

• if the mother:

has reached the local legal age for participating in medical research studies

is HIV-1 infected (with or without HIV-2 infection) and is not eligible for HAART

has received some antiretroviral prophylaxis during pregnancy or delivery,

has an antenatal lymphocyte CD4 count above the threshold for HAART initiation in pregnant women according to the national recommendation in each site

resides within the study area and is not intending to move out of the area in the next year

gives consent to participate for her and her infant

A baby will be excluded if:

• S/he presents clinical symptoms and/or biological abnormalities equal to or greater than grade II of the ANRS classification for adverse event on the day of enrolment, with exceptions for haemoglobin (baby excluded if Hb < 12 g/dL) and absolute neutrophils count (baby excluded if neutrophils < 1200 cells/μL)

• S/he presents with serious congenital malformation(s)

• Her/his birth weight was < 2.0 kg

• The mother has participated in the ANRS 12174 trial for a previous pregnancy

• S/he and her/his mother are participating in another clinical trial on the day of enrolment

### Main study outcomes

#### Primary outcome measures

• Acquisition of HIV-1 (as assessed by HIV-1 DNA PCR) between day 7 and 50 weeks of age.

#### Secondary outcome measures

• Severe adverse events (SAE) grade III or IV possibly-related or with undetermined relation to the study drug (according to ANRS SAE reporting procedures), up to 50 weeks.

• In vitro HIV-1 resistance to antiretrovirals in infants who will get infected with HIV-1 during the study

• Growth, i.e. length and weight up to 50 weeks of age

• HIV-1-free survival from day 7 until 50 weeks (event: infant death or acquisition of HIV-1 infection in infants)

HIV acquisition is defined as follows:

##### During the course of the trial

HIV-1 infection will be established if HIV-1 DNA is detected in blood (detection threshold of 150 viral copies/mL). All first positive HIV-1 DNA PCR will be confirmed by a second test on another sample taken 2 to 5 days later. If the second test is negative, then the child will be considered HIV-1 uninfected for this study visit.

##### At enrolment (day 7)

In order to ensure that infants infected with HIV-1 at week 6 (or at first follow-up visit if week 6 visit is missed) were not already infected at day 7, we will systematically and retrospectively re-test their dried blood spots (DBS) collected at day 7 using both a Roche-Amplicor HIV-1 DNA PCR and a real time HIV-1 RNA PCR (Figure 1). In case of a positive result with at least one of the latter tests, the child will be considered HIV-1 infected at day 7 and therefore excluded from the trial. This situation is expected to occur for very few children.

### Randomisation and stratification

Infants fulfilling the inclusion criteria will be randomised on day 7 (+/− 2 days). Randomisation will be stratified by country using permuted blocks of randomly varying sizes 4 and 6. The eligible infants will be allocated to one of two arms: LPV/r (twice daily) or Lamivudine (twice daily) according to a 1:1 ratio. All randomisation lists will be prepared (and kept) by an independent statistician in Montpellier, as well as by the data monitoring committee (DMC) statistician.

### Screening, Follow-up visits and outcome measurements

#### Antenatal screening (screening 1)

All antenatal clinics where the women will be screened benefit from a PMTCT programme including routine counselling and HIV testing. All HIV-infected pregnant women attending these ANCs with a gestation period between 28 and 40 weeks will be targeted for the first screening visit. After comprehensive counselling on infant feeding, mothers intending to breastfeed will be briefly informed about the study and invited to attend the first screening visit. After informed consent for this visit, women will be offered a CD4 cell count and clinical assessment (HIV-1 disease staging and investigation for opportunistic infections). All HIV-1-infected women and their babies will access PMTCT perinatal prophylaxis according to national guidelines [23]. Pregnant women will be encouraged to deliver at a maternity clinic. Pregnant women who require ART according to existing guidelines [23] will be referred to national ART access programmes in order to receive HAART for the remainder of the pregnancy, through delivery and continued life-long. She and her infant will not be eligible for the trial.

#### Post-natal screening (screening 2)

The post-natal screening will be carried out from 1 to 6 days after delivery. The baby will be tested for HIV-1 infection using a real-time PCR DNA assay, and for biological abnormalities (full blood count, ALAT/ASAT, serum-creatinine). Finally, the study physician will assess the baby for clinical exclusion criteria. If these clinical criteria are met, the baby will be referred to the reference paediatrician for appropriate care.

#### Follow-up procedures

**Figure 2:**
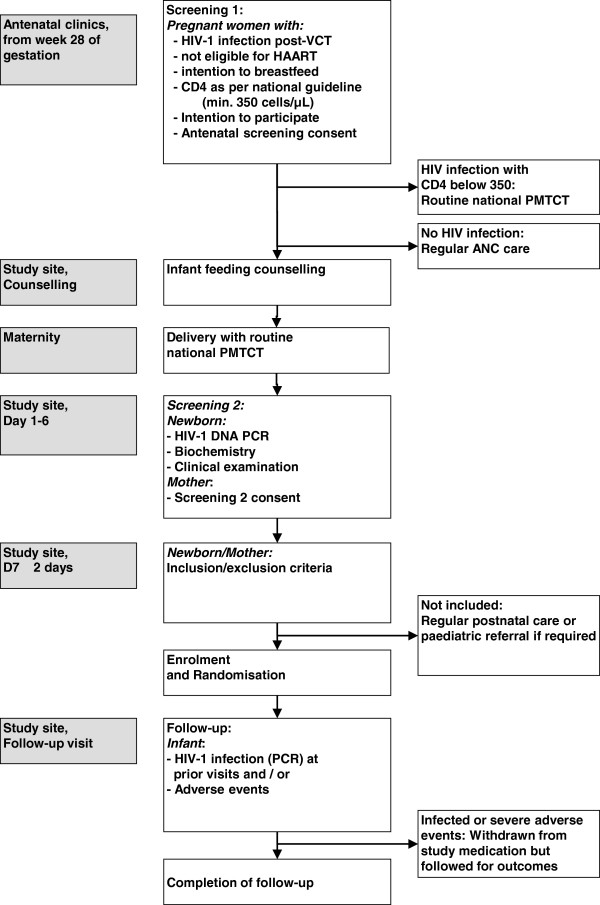
ANRS 12174 PROMISE-PEP trial screening, enrolment and follow-up visits contents.

Systematic follow-up visits will be organised at week 2 and every 4 weeks until week 50 (final visit) (Figure 2), with clinical examination, adverse event reporting, infant length/weight, counselling on infant feeding and drug adherence. The primary outcome (HIV-1 DNA) will be measured at week 6, 14, 26, 38 and 50.

Mothers and babies will be followed by research/clinic-based visits, and if needed, by community-based visits. Children will be assigned to scheduled clinic visits and mothers will be encouraged to come back to the research site whenever they or their babies are sick.

In babies, adverse events will be searched for and reported. Severe adverse events will be reported according to the sponsor guidelines. Infant feeding practices will be monitored using tools based on WHO guidelines [24]. Weight and length will be measured twice based on guidelines developed by the WHO and z-scores calculated using the new WHO child growth standards [25] and the free WHO program ( http://www.who.int/childgrowth/training/en/). The intervention will be stopped in children shown to be HIV-1-infected in order to reduce the period of unnecessary exposure to LPV/r or Lamivudine mono-prophylaxis and risk of emergence of resistant viral populations to a minimum. At 50 weeks of age, a final real time DNA PCR test will be done on all babies, in order to detect late HIV-1 transmission due to prolonged or resumed BF. Child survival will be assessed at 38 weeks and at 50 weeks of age. Hospital records and verbal autopsy will be used to measure overall and cause-specific infant mortality. Morbidity will be measured by recall within the last week of the visit, in-between visits and hospitalisations since last visit.

### Study treatment

The study treatments will be administered using commercial syrup formulations of lopinavir/ritonavir (80/20 mg/mL) or lamivudine (10 mg/mL). Study drug bottles of LPV/r and Lamivudine will be masked by study labels.

Lopinavir/ritonavir (80/20 mg/mL) will be given to the baby twice daily as recommended in infants, with the following scheme: 40/10 mg (0.5 mL) twice daily if 2-4 kg and 80/20 mg (1 mL) twice daily if >4 kg. Similarly, lamivudine will be given as follows: 7,5 mg (0,75 mL) twice daily if 2-4 kg, 25 mg (2.5 mL) twice daily if 4-8 kg and 50 mg (5 mL) twice daily if >8 kg. These dosages were calculated on the basis of a pharmacokinetic study of LPV/r in young infants done in Paris [21]. For Lamivudine, calculations were based on a pharmacokinetic study carried out by Moodley et al. [26]. Infants whose mothers need and initiate HAART during the trial will be discontinued from the study medication in order to avoid excessive intake of ART in babies due to high diffusion of lamivudine in breast milk [27]. In addition, the maternal HAART regimen will much decrease the risk of HIV transmission through breast milk [9].

### Study size

The primary outcome will be assessed using a superiority analysis. We expect the risk of acquiring HIV-1 infection between day 7 and 50 weeks of age to range between 3% and 5% in the lamivudine group, based on the findings of the MITRA study which reported a transmission of 1.2% (95%CI: 0 to 2.4) between 6 weeks and 6 months and association between CD4 count and risk of transmission observed in the VTS study [2]. This range of 3 to 5% accounts for population with CD4 count > 350 cells/μl and additional HIV transmission between day 7 and week 6, and between 6 months and 50 weeks.

Considering its higher anti-HIV-1 activity, we expect a higher efficacy of LPV/r, ranging from 1 to 2.5%, which would be considered as a clinically relevant difference compared with the lamivudine arm.

Table 1 displays different scenarios, based on a comparison of proportions with 80% power, a two-sided alpha error of 5% and 10% lost to follow-up.

Including 1,500 infants in the trial (750 in each arm) would allow us to cover most scenarios under the above hypotheses.

### Data analysis

**Figure 3:**
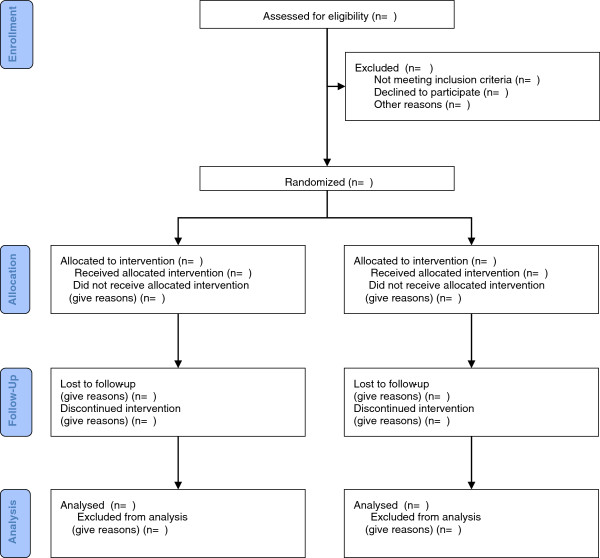
ANRS 12174 PROMISE-PEP trial, planned CONSORT patient flow chart.

Analysis methods will follow the CONSORT guidelines [28] (Figure 3) and recommendations of the GHENT group related to the mother-to-child transmission studies [29,30] and breastfeeding patterns [31].

All tests will be two-sided. Descriptive results, efficacy and safety estimates and their corresponding 95% CIs will be presented. The statistical significance is set at p < 0.05.

Provided there are no major differences of relative transmission risk between sites, the stratified (site-specific) randomisation will be adjusted for in the analyses. Moreover, potential confounders, such as maternal CD4 counts, will be considered for further adjustment if they are imbalanced at baseline, according to thresholds pre-defined in the plan of analysis.

#### Interim analysis

One interim analysis will be carried out by the DMC when about half of the projected HIV-transmissions have occurred. We will suggest that the DMC follows the DAMOCLES group recommendations [27].

#### Primary analyses

Analyses for the primary endpoint will be undertaken on an intention-to-treat basis.

– Uninfected drop-outs and deaths will be censored at the last outcome measurement.

– Children of mothers initiating HAART during the study will no longer receive the study intervention. They will be followed up for all outcomes as the other children and will not be censored in the primary analysis.

– Children who stop breastfeeding for various reasons (mother illness or death, etc.) will be withdrawn from study drugs one week later. They will continue follow-up until 50 weeks of age and will be included in the primary analysis.

##### Primary endpoint

The main endpoint is acquisition of HIV-1 (as assessed by HIV-1 DNA PCR) between day 7 and 50 weeks of age, as defined previously.

The primary endpoint will be analysed using a time to event approach. HIV-1 infections occurring between day 7 and week 50 will be compared between the two groups of treatment using proportional hazard modelling with hazard ratio and its 95% CI adjusted for site and any confounders.

Relative treatment efficacy will be calculated as 100 × (1-HR) where HR is the hazard ratio of HIV-1 transmission in the LPV/r group compared to the Lamivudine group.

The effect size will also be expressed as the absolute difference between risks with its 95% confidence interval. The reduction in the number of patients who need to be treated when given LPV/r compared to when given Lamivudine to prevent HIV-1 infection in one infant will be computed [32].

#### Secondary analyses

If LPV/r is not demonstrated to be superior to Lamivudine in the primary analysis, we will conduct a non-inferiority analysis of the primary outcome.

This analysis is supported by the expectancy of a better resistance profile of LPV/r compared with Lamivudine. A 95% confidence interval (adjusted for interim analyses) for the hazard ratio of the primary efficacy outcome will be constructed. The upper boundary for the non inferiority is set at < 1.2, as we believe that any further reduced efficacy would have important public health consequences.

We will also carry out per protocol analyses for this non-inferiority analysis. The conclusion will be drawn from both the intention to treat and the per protocol analyses.

Under the closed-testing procedure with sequential testing of the superiority and non-inferiority analyses, the overall 2-sided type 1 error is maintained at 5%.

### Laboratory assays

All biological specimens will be analysed and stored at each study site.

Paediatric HIV-1 infection will be diagnosed at each site on Dried Blood Spots (DBS) by means of real-time PCR, using a commercial kit (Generic HIV Charge Virale, Biocentric, France) [33]. When necessary (see Figure 1), a commercial real time RNA PCR (Generic HIV Charge Virale, Biocentric, France) as well as another commercial DNA PCR test (Roche Amplicor) will be used in the coordinating laboratory (Montpellier). The real-time PCR technique is implemented with the support of well-validated, standardised protocols designed by a working group of the French National Agency for Research on AIDS and Viral Hepatitis (ANRS) [34]. It is well adapted to the use of DBS collected on filter paper, an ideal tool for early paediatric HIV-1 diagnosis [35].

Maternal HIV-1 antibody detection will be performed by ELISA and/or rapid tests, according to each site’s national optimised algorithms [36]. Maternal plasma HIV-1 RNA quantification (“viral load”) will be measured by means of a commercially available real time RNA PCR test (Generic HIV Charge Virale, Biocentric, France) [33]. CD4 counts will be performed by flow cytometry. Haematological and biochemical assays will be performed on babies' plasma samples by means of already available automated procedures at the site laboratories. These tests will be carried out for safety monitoring, and will include full blood counts and blood concentration of liver enzymes. In addition, plasma LPV/r and Lamivudine will be detected in blood at weeks 6 and 38 by high pressure liquid chromatography (HPLC) (JM Tréluyer, Saint-Vincent de Paul Hospital, Paris).

HIV resistance mutations to antiretrovirals will be identified by sequencing of the pol genes.

Laboratory and quality control procedures will follow WHO Good Laboratory Practice Guidelines [37].

HIV-1 serology, CD4 counts, and haematology/biochemistry assays will be submitted to a stringent quality assessment programme, to an international external quality control programme and to intensive GLP training addressed to the laboratory personnel in all 4 sites.

#### Training, technology transfer and standardisation

Real time PCR technology transfer and training will be implemented in all 4 sites with implementation of a quality assessment and quality control programme from the Montpellier team and the ANRS AC11 medical virology group. Ongoing training and monitoring will occur during the full duration of the follow-up period of the study. Likewise, initial and ongoing training and standardization will be undertaken to ensure adequate reproducibility and validity of anthropometric measurements.

### Human subjects considerations

The study will be conducted in accordance with the sponsor’s (ANRS) Charter of Ethics, with the International Conference on Harmonisation (ICH) guidelines for good clinical practice and with the Medicines Control Council guidelines for good clinical practice in the conduct of clinical trials in human participants in South Africa. The study protocol has been submitted to and approved by the Ethical Committee for Health Research in Burkina Faso, the Biomedical research Ethics Committee in Zambia, the Uganda National Council for Science and Technology, the Stellenbosch University ethical committees, the Medicines Control Council in South Africa and the Regional Committee for Medical research Ethics of Norway.

Written informed consent and assent will be obtained in vernacular language from the mother of participating mother-infant pairs. The informed consent process will be implemented with an independent third person (the ‘witness’) for illiterate mothers or when translation is required. This witness will sign the consent form together with the mother and the investigator.

Mothers requiring HAART during pregnancy and infants diagnosed with HIV at screening or during the trial will be referred to the study-related HIV care unit for prompt HIV management according to local guidelines. In particular, infants will be treated with HAART as soon as HIV infection is diagnosed.

## Discussion

This trial will compare the efficacy and safety of infant lopinavir/ritonavir versus lamivudine to reduce HIV-1 transmission during breastfeeding. Although NVP was studied more extensively than Lamivudine for PEP purposes, we decided to use the latter drug for the comparative arm as we expect a similar efficacy as NVP (according to the MITRA study) with similar adverse events but with less detrimental genetic barrier profile. Indeed, we believe that the ‘cost’ of using NVP for PEP during breastfeeding (i.e. to deprive infants who will fail PEP of a major class of ART for their HIV treatment) is too high to recommend this drug for prevention of postnatal HIV transmission.

Active clinical and biologic follow up of infants and systematic report of potential effects will be organised according to GCP and GLP international guidelines for toxicity and adverse event management. Recently, breastfed infants of HAART-treated mothers have been shown to ingest sufficient amounts of ART by breastfeeding to reach plasma therapeutic levels (at least for NVP, AZT and 3TC) [22]. Therefore, in order to avoid additional toxicity and overdosage of lamivudine or LPV/r in babies (and because maternal HAART is most likely to reduce postnatal HIV-1 transmission) our trial will recruit only babies born of mothers not eligible for HAART. Likewise, infants whose mothers need and initiate HAART during the trial will also be discontinued from the study medication. Similar to post-exposure prophylaxis, the study medication will continue one week after cessation of breastfeeding (= last exposure of HIV), after a maximum of 49 weeks of breastfeeding. This extended period of study treatment is justified as it parallels standard post-exposure prophylaxis.

This study will provide a new evidence-based intervention to support HIV-1-infected women not eligible for HAART to safely breastfeed their babies, thus counteracting the existing contradiction between optimal infant feeding and PMTCT through breast milk. Assuming a full coverage and 60% efficacy of the intervention, about 120,000 cases of post-natal HIV-1 transmission could be averted per year in Africa. In addition, a prolonged breastfeeding period of HIV-infected mothers will reduce stigmatisation of infant feeding patterns and disclosure of maternal HIV status [38], and will improve health outcomes in infants born to HIV-infected mothers. By extension, this intervention will also contribute to promote breastfeeding in the communities beyond HIV-infected women [8,39]. Optimised breastfeeding promotion has the potential of annually averting 1.3 million child deaths globally, if universally implemented.

## Competing interests

The authors declare that they have no competing interests.

## Authors’ contributions

TT and PV designed the study. All authors contributed to the study design and the protocol writing. NN drafted this manuscript and all co-authors provided constructive comments. All authors read and approved the final manuscript.

## Authors’ information

PROMISE-PEP Study group

University of Montpellier 1 (France): Philippe Van de Perre (principal investigator), Nicolas Nagot (project leader), Roselyne Vallo (central data manager), Valerie Marechal (central lab coordinator), Dorine Neveu (statistician), Vincent Foulongne (virologist), Michel Segondy (virologist), Roxanne Schaub (statistician)

University of Paris V (France): Stephane Blanche (paediatrician), Jean-Marc Treluyer (pharmacologist), Deborah Hirt (modeler)

Makerere University (Uganda): James K Tumwine (Site PI), Charles Karamagi (Investigator), Philippa Musoke (Investigator), Grace Ndeezi (Invesitgator), Proscovia M Mugaba, Mary Kwagala (Coordinator), Joan Murungi (Clinician), Hawa Nabuuma Muweesi (Lab Coordinator), Evelyn Ninsiima (Lab Technologist), Simon Baryeija (Pharmacist)

University of Ouagadougou (Burkina Faso): Nicolas Meda (site principal investigator), Rasmata Ouédraogo (biologist), Diarra Yé (paediatrician), Eric Somé (site trial coordinator), Hugues A. Traoré (site clinical study monitor), Christelle Nadembega (site biological study monitor), Justin Konaté (assistant of biological study monitor), Arsène Zongo (site pharmacist), Abass Ouédraogo (pharmacist assistant), Désiré Néboua (study physician), Aissatou Bélemviré (study physician), Armel Bambara (site data manager), Justine Boncoungou (social worker), Danielle Zoungrana (social worker)

University of Western Cape (South Africa): Cheryl Nikodem (Site principal investigator), Kim Harper (Site Co-principal investigator), Debra Jackson (Co-investigator), David Sanders (Country Principal Investigator), Mandisa Singata (project leader), Amwe Sunday (Research clinician), Collins Okegbe-Eze (Research clinician), Xoliswa Williams (Research clinician), Nolundi Mshweshwe (Research clinician), Vatiswa Henge (Pharmacist), Fikiswa Gomba (Breastfeeding counsellor), Lada Nikodem (Data manager), Oswell Khondowe (Trainer)

University of Zambia (Zambia): Chipepo Kankasa (Site principal investigator), Mwiya Mwiya (site trial coordinator), Mildreed Lusaka (UTH Site Coordinator), Mary Chizyuka (UTH Site co-Coordinator), Mary Phiri (Chawama Site Coordinator), Billies Imakando (Chawama Site Coordinator), Mwenechanya Musaku (study physician), Monica Kapasa (study physician), David Rutagwera (Laboratory Coordinator), Ngondwe Clement (co-Laboratory Coordinator), Hilton Mwila Mwaba (lab scientist), (lab scientist), Japhet Matoba (Laboratory Scientist), Hilton Mwaba (Laboratory Technician), Chafye Siumita (Adminstrator/Data Clerk), Katai Chola (Data Manager), Patricia Mwamutanda (Pharmacist).

University of Bergen (Norway): Thorkild Tylleskar (Site principal investigator), Halvor Sommerfelt (investigator), Ingunn Engebretsen (investigator), Jørn Klungsøyr (investigator), Jan van den Broeck (investigator).

## Pre-publication history

The pre-publication history for this paper can be accessed here:

http://www.biomedcentral.com/1471-2334/12/246/prepub
